# Oligogalacturonides: plant damage-associated molecular patterns and regulators of growth and development

**DOI:** 10.3389/fpls.2013.00049

**Published:** 2013-03-13

**Authors:** Simone Ferrari, Daniel V. Savatin, Francesca Sicilia, Giovanna Gramegna, Felice Cervone, Giulia De Lorenzo

**Affiliations:** Dipartimento di Biologia e Biotecnologie “Charles Darwin”, Sapienza Universitàdi Roma, Rome, Italy

**Keywords:** oligogalacturonides, damage-associated molecular patterns, innate immunity, pectin, elicitors, signal transduction, defense responses, cell wall

## Abstract

Oligogalacturonides (OGs) are oligomers of alpha-1,4-linked galacturonosyl residues released from plant cell walls upon partial degradation of homogalacturonan. OGs are able to elicit defense responses, including accumulation of reactive oxygen species and pathogenesis-related proteins, and protect plants against pathogen infections. Recent studies demonstrated that OGs are perceived by wall-associated kinases and share signaling components with microbe-associated molecular patterns. For this reason OGs are now considered true damage-associated molecular patterns that activate the plant innate immunity and may also be involved in the activation of responses to mechanical wounding. Furthermore, OGs appear to modulate developmental processes, likely through their ability to antagonize auxin responses. Here we review our current knowledge on the role and mode of action of this class of oligosaccharides in plant defense and development.

## INTRODUCTION

The first evidence that pectin fragments induce defense responses was provided more than 30 years ago by assaying phytoalexin accumulation in soybean cotyledons (Hahn et al., 1981). These fragments, called endogenous elicitors, were later identified as oligomers of alpha-1,4-linked galacturonosyl residues (oligogalacturonides, OGs) that can be obtained by partial hydrolysis of polygalacturonic acid (Nothnagel et al., 1983). It was speculated that the degradation of a major component of pectin, i.e., homogalacturonan (HGA), which occurs during microbial infections, may cause the accumulation of OGs that trigger defense responses. Around that time, it was shown that digestion of HGA by a fungal or a tomato-derived polygalacturonase (PG) releases an elicitor of a wound-inducible proteinase inhibitor (PI), suggesting a role of OGs in the wound response (Bishop et al., 1981). A few years later it was reported that OGs antagonize the activity of auxin during pea stem elongation, envisioning a possible role of these oligosaccharides as regulators of growth and development (Branca et al., 1988). In subsequent years, efforts were made to elucidate the mechanism of action of OGs and to investigate their ability to trigger plant defenses as well as to affect, as local antagonists of auxin, plant growth and development. However, only recently significant progress has been made in understanding the basis of OG perception and signal transduction. Here we review our current knowledge on the effects and mode of action of OGs in plant defense and development.

## OGs ARE ELICITORS OF DEFENSE RESPONSES

Pathogens need to be recognized in a timely manner by the host in order to activate the proper defenses that restrict invasion and colonization. A crucial feature of the innate immune system in both plants and animals is the ability to sense a potential danger through the recognition of molecules that alert the cell. Molecules associated with pathogenic microbes (microbe-associated molecular patterns, MAMPs), like chitin from fungi, peptidoglycan and flagellin from bacteria and glucans from the cell wall of oomycetes are specifically sensed by the host cells and trigger an immune response (Bittel and Robatzek, 2007; Boller and Felix, 2009). MAMP-triggered immunity is now a fertile field of research in plant biology.

**FIGURE 1:**
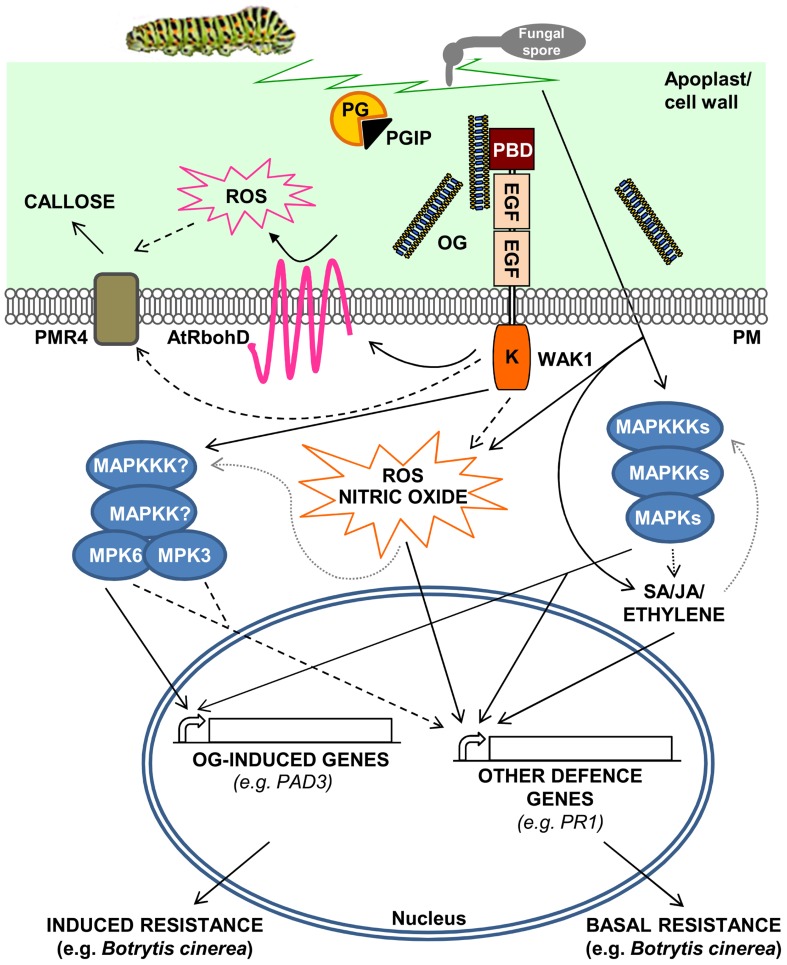
**A model for the activation of *Arabidopsis thaliana* defense responses triggered by oligogalacturonides (OGs).** OGs are released from the cell wall after degradation of homogalacturonan by mechanical damage or by the action of hydrolytic enzymes such as PGs, secreted by pathogens. PGIPs in the apoplast modulate PG activity, favoring the accumulation of elicitor-active OGs, which function as DAMPs. OGs are perceived by WAK1 and trigger defense responses such as ROS accumulation through the activation of the NADPH oxidase AtRbohD, nitric oxide production, callose deposition, and MAPK-mediated activation of defense gene expression. Pathogen invasion or mechanical damage also cause an increase of JA, SA, and ethylene levels, mediated by MAPK cascades, triggering defense responses independently of OGs. DAMP- and hormone-mediated defense responses result, respectively, in induced and basal resistance toward necrotrophic pathogens, such as *Botrytis cinerea*. Dashed lines indicate hypothetical cascades; dotted gray lines indicate oversimplification of the complex and still partially uncharacterized roles of MAPKs in the regulation of hormone and ROS synthesis/response.

Response to endogenous signals originating from stressed or injured cells, the so-called “regulation from within,” is now emerging as an important function of the immune system. Endogenous molecules with elicitor activity are released from cellular components during pathogen attack or abiotic stresses, and have been indicated as damage-associated molecular patterns (DAMPs) in both plants (Boller and Felix, 2009; Galletti et al., 2009; Tor et al., 2009; De Lorenzo et al., 2011; Ranf et al., 2011) and animals, where they have also been called alarmins (Bianchi, 2007; Lotze et al., 2007). OGs are probably the best characterized plant DAMPs and elicit in several plant species a wide range of defense responses, including accumulation of phytoalexins (Davis et al., 1986), glucanase, and chitinase (Davis and Hahlbrock, 1987; Broekaert and Pneumas, 1988), deposition of callose, production of reactive oxygen species (ROS; Bellincampi et al., 2000; Galletti et al., 2008), and nitric oxide (Rasul et al., 2012; **Figure 1**). OGs are thought to be released from plant cell walls upon partial degradation of HGA by microbial PGs during infections (Cervone et al., 1989) or by the action of endogenous PGs induced by mechanical damage (Orozco-Cardenas and Ryan, 1999). The signaling activity of OGs is a clear indication that plants have evolved mechanisms to monitor HGA degradation for the early detection of tissue injury. Pectin is one of the most accessible components of the cell and, therefore, is among the first structures to be altered during an attempted pathogen invasion or when the wall undergoes a stress rupture (De Lorenzo and Ferrari, 2002). Since plant cell wall integrity may be efficiently watched by monitoring the pectin status, we have proposed the existence of a system, called “pectin integrity monitoring system” or PIMS, dedicated to this function (De Lorenzo et al., 2011). OGs are likely located in a key position in PIMS, that allows them to act as indicators of cell wall integrity, both in adverse conditions and during normal growth (see below). Moreover, because HGA-degrading enzymes such as PGs are among the first enzymes secreted by microbes during host colonization, PIMS also includes the inhibitors of fungal and insect PGs (PG-inhibiting proteins or PGIPs), which guard the cell wall by limiting HGA degradation (De Lorenzo et al., 2001; De Lorenzo and Ferrari, 2002; Di Matteo et al., 2006). By inhibiting the action of PGs secreted by pathogens, PGIPs not only hinder pectin degradation, but also favor the accumulation of elicitor-active OGs (De Lorenzo et al., 1994, 2001; De Lorenzo and Ferrari, 2002), thus playing a dual role in PIMS.

A structural requirement for the biological activity of OGs is a degree of polymerization (DP) between 10 and 15 (Côté and Hahn, 1994). This size is optimal for the formation of Ca^2^^+^-mediated intermolecular cross-links resulting in structures called “egg boxes” (Braccini and Perez, 2001; Cabrera et al., 2008), that are thought to be necessary for OG activity. Modification of the reducing end of OGs does not affect the formation of egg boxes (Cabrera et al., 2008) and does not affect elicitor activity (unpublished results of our lab). OGs with a DP of 2–6, which we indicate as short OGs, have been reported in a few cases to exhibit elicitor activity as, for instance, during the induced expression of PIs in tomato (Farmer and Ryan, 1990; Moloshok et al., 1992); however, short OGs appear to suppress defense responses in wheat (Moerschbacher et al., 1999). HGA is synthesized in an esterified form in the Golgi apparatus and, subsequently, is secreted into the cell wall where it undergoes a partial de-esterification by the action of pectin methylesterases (PMEs; Pelloux et al., 2007). The degree of esterification of HGA varies in different tissues according to the specific developmental stage (Wolf et al., 2009); consequently OGs with different degrees of esterification are expected to be released under diverse circumstances. In most studies, OGs have been prepared from polygalacturonic acid digested with commercial PGs (Nothnagel et al., 1983; Galletti et al., 2008; Cabrera et al., 2010), and it is not yet clear how esterification affects their biological activity. Acetylated OGs, but not de-esterified OGs, reduce the haustoria formation of *Blumeria graminis* growing on wheat leaves, suggesting that esterification is necessary for some specific responses (Pelloux et al., 2007; Randoux et al., 2010). The presence of OGs with a low degree of methylation in strawberry fruits overexpressing a PME was correlated with the expression of defense responses and with a concomitant partial resistance against *Botrytis cinerea* (Osorio et al., 2008). On the other hand, *Arabidopsis thaliana* plants overexpressing an inhibitor of PME or mutated in an endogenous PME have a high degree of pectin methylesterification (Lionetti et al., 2007, 2010; Raiola et al., 2010, 2011). These plants do not show constitutive expression of defense responses but, nevertheless, exhibit enhanced resistance to *Botrytis cinerea* and *Pectobacterium carotovorum.* A reduced growth of these pathogens on highly methylated pectin as a carbon source may in part explain the resistant phenotype of these plants (Lionetti et al., 2012).

## DISSECTION OF OG SIGNALING

Until recently, the effects of OGs were studied in non-model plants, such as soybean (Hahn et al., 1981), and therefore it was difficult to identify the molecular components responsible for their perception and transduction. An additional limitation of these studies was that OG production and accumulation is hard to detect *in vivo*, unless in the presence of a massive tissue degradation that generally occurs only during the later stages of plant infections (An et al., 2005). The adoption of *Arabidopsis* as a model plant has provided a useful tool to advance our knowledge of the OG biology. Notably, the responses triggered by OGs in *Arabidopsis* largely overlap those activated by MAMPs. For instance, transcript profiling of seedlings treated with either OGs or flg22, i.e., a peptide that comprises the active epitope of flagellin (Gomez-Gomez et al., 1999), indicates an extensive overlap of responses, at least at the early times after treatment (30–60 min; Denoux et al., 2008). In *Arabidopsis*, both elicitors activate a set of responses that are independent of the signaling pathways involving ethylene, salicylic acid (SA), and jasmonate (JA; Zipfel et al., 2004; Ferrari et al., 2007) and induce the phosphorylation of two mitogen-activated protein kinases (MAPKs), namely AtMPK3 and AtMPK6 (Denoux et al., 2008; Galletti et al., 2011). AtMPK6 appears necessary for the early expression of defense genes and for the induced resistance against *Botrytis cinerea* triggered by these elicitors (Galletti et al., 2011). Furthermore, both OGs and flg22 trigger a robust oxidative burst mediated by the nicotinamide adenine dinucleotide phosphate (NADPH) oxidase AtRbohD, which is at least partially responsible for the subsequent production of callose (Zhang et al., 2007; Galletti et al., 2008) by the callose synthase POWDERY MILDEW RESISTANT 4 (Nishimura et al., 2003; **Figure 1**). However, OGs are relatively weak elicitors compared to flg22, probably as a consequence of their reduced half-life (Denoux et al., 2008). For instance, flg22 and other MAMPs, in contrast to OGs, induce the expression of defense genes dependent on SA, JA, and ethylene signaling, such as the well characterized SA-dependent marker gene *PR-1* (Denoux et al., 2008). These additional defense responses likely contribute to basal resistance to pathogens. Moreover, OGs are endogenous signals likely released in low amounts also in healthy plant tissues, as a consequence of developmentally related cell wall remodeling processes. Whether plants can discriminate between low physiological doses and higher amounts of OGs produced in pathological situations has not been elucidated yet. Intriguingly, a mutual interference has been observed between responses induced by flg22 and OGs, suggesting that they differ not only from a quantitative, but also from a qualitative point of view (Aslam et al., 2009).

Understanding how OGs are perceived is necessary to elucidate their role *in vivo*, but the identification of an OG receptor has been daunting for a long time. Wall-associated kinases (WAKs) were indicated as interesting candidates because of their ability to bind OGs and polygalacturonic acid (Anderson et al., 2001; Decreux and Messiaen, 2005). WAKs are receptor-like kinases, with an extracellular domain containing epidermal growth factor motifs, a transmembrane domain and an intracellular Ser/Thr kinase domain (Anderson et al., 2001). *Arabidopsis* has a small family of five *WAK* genes and a larger family of 22 *WAK-like* (*WAKLs*) genes (Verica et al., 2003), though in monocots these families appear largely expanded (Zhang et al., 2005). WAKs were first identified in *Arabidopsis* as pectin-bound proteins, since only harsh treatments, i.e., boiling in the presence of high concentrations of detergents and reducing agents or pectinase digestion could solubilize a protein reacting with an anti-WAK polyclonal antibody (He et al., 1996; Lally et al., 2001; Wagner and Kohorn, 2001). The same band of about 68 kDa (lower than the theoretical 78 kDa mass of the mature WAK1, which contains eight predicted glycosylation sites), also reacted with monoclonal antibodies against partially esterified HGA (Wagner and Kohorn, 2001). This led to the conclusion that WAK1 is tightly bound to pectin. Subsequently, WAK1 was shown to carry a N-terminal pectin binding domain that interacts with non-methylesterified HGA and OGs in a Ca^2^^+^-dependent manner (Decreux and Messiaen, 2005). Notably, OGs with DP > 9 (i.e., those with elicitor activity) bind reversibly to WAK1 and binding increases when OGs are present as dimers in a calcium-mediated “egg box” conformation (Cabrera et al., 2008). Decreux et al. (2006), using site-directed mutagenesis, also identified five basic amino acids in the WAK1 ectodomain that are involved in the binding to HGA dimers and multimers. The ectodomains of WAK1 and WAK2 bind de-esterified HGA but not highly esterified HGA or other structurally different pectic components, such as rhamnogalacturonan I (RGI) and rhamnogalacturonan II (RGII; Kohorn et al., 2009). This behavior is at odds with the observation that leaf mesophyll protoplasts from a *wak2* knock out (KO) mutant are unable to induce the expression of a vacuolar invertase gene upon treatment with either de-esterified and esterified HGA, RGI, and RGII (Kohorn et al., 2009). Whether *wak2* protoplasts have reduced responsiveness specifically to pectin and not to other non-pectic polysaccharide signals, such as chitin or chitosan, was not verified. These data are difficult to be taken as an evidence that WAK2 is a receptor for pectin: given the size and the extreme structural complexity of pectin, proposing its interaction with WAKs as a classical receptor–ligand interaction without pointing to a specific structural domain as a target for recognition, can be misleading (Kohorn and Kohorn, 2012). Many observations, instead, indicate a specificity or at least a preference of WAK1 and WAK2 for de-esterified HGA and for OGs (Decreux and Messiaen, 2005; Kohorn et al., 2009). The hypothesis of WAKs as receptors of OGs has been difficult to test through conventional genetic approaches due to functional redundancy. In particular, *Arabidopsis* KO mutants for individual *WAK* genes do not show a significant altered OG responsiveness (unpublished results), and generation of double or multiple mutants is difficult because the genes are tightly clustered (Verica et al., 2003). A chimeric receptor approach, however, revealed that WAK1 acts as a receptor of OGs (Brutus et al., 2010). The extracellular domain of WAK1 was fused with the kinase portion of EF-Tu receptor (EFR), the receptor of the bacterial MAMP elf18 (Zipfel et al., 2006), and the chimeric receptor was able to activate the kinase domain in response to OGs. On the other hand, upon stimulation with elf18, a chimeric receptor formed by the EFR ectodomain and the kinase domain of WAK1 activated the typical responses triggered by OGs.

Both pectin and OGs affect plant gene expression, but the induced transcriptional profiles appear different. For instance, the expression of about 50 genes is up-regulated in *Arabidopsis* protoplasts treated with high molecular weight pectin (Kohorn et al., 2009), whereas the expression of more than one thousand genes is induced in *Arabidopsis* seedlings treated with OGs (Ferrari et al., 2007; Denoux et al., 2008). Notably, very few of the genes up-regulated by pectin treatments are also induced by OGs in seedlings. Probably, pectin and OGs trigger different responses, and more than one receptor is involved in their perception.

The analysis of the expression of WAKs and WAKLs suggests that most of them play a role in defense. Public microarray data indicate that *WAK1*, *WAK2*, and* WAK3 *are all induced by *Phytophthora parasitica*, ozone and benzothiadiazole, an activator of the systemic acquired resistance, while *WAK1* and *WAK2* are also induced by SA. Interestingly, *WAK1* is induced in *Arabidopsis* seedlings by OGs, but, unexpectedly, none of the *WAKs* is up-regulated by flg22 (Denoux et al., 2008). Overexpression of *WAK1 *in *Arabidopsis* or of the rice *OsWAK1* increases resistance to *Botrytis cinerea* and *Magnaporthe oryzae*, respectively (Li et al., 2009; Brutus et al., 2010); the *Arabidopsis* *WAKL22* confers resistance to *Fusarium oxysporum *(Diener and Ausubel, 2005), though its mode of action is not known.

The role of WAKs under physiological conditions is less clear. Since pectin is tightly linked to the extracellular domain of WAKs, these proteins are good candidates to monitor pectin integrity. Indeed, reduced expression of WAKs through inducible antisense constructs causes reduced growth, suggesting a role of these proteins in regulating cell expansion (Lally et al., 2001; Wagner and Kohorn, 2001). This is in agreement with the observation that *wak2* mutants have reduced growth only in the absence of osmotic support, suggesting that they have a defect in cell wall structure and/or composition (Kohorn et al., 2006).

The perception of non-self molecules or of a damaged self is a taxonomically widespread mechanism, and may have an adaptive significance. Striking parallels exist between pectin/OGs and hyaluronan/hyaluronan fragments found in animal cells. The breakdown of hyaluronan, a component of the animal extracellular matrix, upon tissue injury or pathogen infection, activates the vertebrate innate immune system (Jiang et al., 2011). Hyaluronan fragments are sensed by the plasma membrane leucine-rich repeat receptors Toll-like receptor 2 (TLR2) and TLR4 (Taylor et al., 2007), which are also required for the perception and activation of the immune response by MAMPs (Scheibner et al., 2006). It is therefore clear that both plants and animals have evolved analogous systems to activate the innate immunity in response to both MAMPs and DAMPs.

## OGs ACT AS SIGNALS IN THE WOUND RESPONSE

Recognition of OGs is likely a crucial function of the plant PIMS, under both physiological and pathological circumstances. One of the most common dangers faced by plants is wounding, as the injured tissue represents an easy entry point for pathogen colonization. Plants are able to sense mechanically damaged tissues as an altered self and respond by activating localized defenses similar to those activated by pathogen infection. These include a rapid oxidative burst (Bradley et al., 1992; Brisson et al., 1994), the expression of defense-related genes (Reymond et al., 2000) and the accumulation of pathogenesis-related proteins (Chang et al., 1995; **Figure 1**). Several genes affected by wounding are also regulated in response to pathogens (Reymond and Farmer, 1998; Durrant et al., 2000; Reymond et al., 2000). A study on the local and systemic wound-induced accumulation of PIs in tomato led to the discovery of systemin, a peptide signal that specifically mediates the systemic wound response, and revealed that also OGs are able to induce PI accumulation (Ryan and Jagendorf, 1995). Therefore, OGs have been hypothesized to be involved in wound signaling, because they can be generated both directly by the physical disruption of HGA and by the action of endogenous PGs (**Figure 1**). Indeed, a tomato PG has been described to be responsible for the production of OGs after wounding (Bergey et al., 1999). However, OGs are likely to act only as local signals, because of their oligoanionic nature and limited mobility in the tissues (Baydoun and Fry, 1985). Their action is independent of systemin: transgenic plants expressing an antisense transcript that decreases systemin levels are defective in the systemic but not in the local expression of PI in response to wounding and normally respond to OGs (McGurl et al., 1992).

Jasmonate is an essential signal in the tomato systemic wound response (Sun et al., 2011), albeit full activation of several JA-regulated defense responses requires ethylene (O’Donnell et al., 1996; Ryan and Moura, 2002). The observations that several wound-responsive genes are JA-independent and that local and systemic wound-induced gene expression are different suggested the existence of two separate signaling pathways in tomato: one mediated by systemin and JA and responsible for the systemic response, the other mediated by OGs but not by JA, and functioning only locally. Cross-talk between the two pathways has been proposed, since OG-induced production of ROS in tomato cells is potentiated by systemin (Stennis et al., 1998). In *Arabidopsis*, like in tomato, both JA and ethylene are required for a stronger and more rapid expression of several wound-responsive genes (Moffat et al., 2012), and local and systemic responses to wounding are different (Rojo et al., 1999, 2003; Delessert et al., 2004). Moreover, also in *Arabidopsis*, OGs up-regulate several wound-responsive genes independently of JA (Leon et al., 2001). However, there are important differences between the wound responses of tomato and *Arabidopsis*. For example, genes encoding systemin are absent in *Arabidopsis*, and JA synthesis is induced by OGs and chitosan in tomato, whereas JA does not accumulate in *Arabidopsis* plants treated with chitosan. In *Arabidopsis*, chitosan blocks JA-induced gene expression through an ethylene-dependent pathway (Rojo et al., 1999). At present, there is no evidence that OGs induce ethylene synthesis (Ferrari et al., 2008; Brutus et al., 2010) and it is not known whether they block JA-induced responses.

Oligogalacturonides protect *Arabidopsis* and grapevine against *Botrytis cinerea *(Aziz et al., 2004; Ferrari et al., 2007). Notably, wounding of *Arabidopsis* induces a strong resistance against the same pathogen (Chassot et al., 2008). Local resistance induced by both OGs and wounding is independent of SA-, JA-, and ethylene-mediated signaling pathways and requires PHYTOALEXIN DEFICIENT 3 (PAD3; Ferrari et al., 2007; Chassot et al., 2008), a cytochrome P450 that catalyses the last step of camalexin biosynthesis (Zhou et al., 1999). Camalexin accumulation is not observed after wounding (Chassot et al., 2008) nor after OG treatment (Ferrari, unpublished results), although priming of camalexin accumulation after inoculation with *Botrytis cinerea* has been observed in wounded leaves (Chassot et al., 2008). These data suggest that wound-induced resistance to *Botrytis cinerea* is mediated by OGs. However, systemic protection against *Botrytis cinerea* is not observed after wounding (Chassot et al., 2008), whereas syringe-infiltration with OGs increases both local and systemic resistance to the fungus (Ferrari et al., 2007), possibly because the amount of infiltrated OGs is higher than that released in the tissue during mechanical damage. It must be also noted that both wounding (Cheong et al., 2002) and OGs (Branca et al., 1988; Bellincampi et al., 1996; Ferrari et al., 2008; Savatin et al., 2011) repress auxin responses (see below), supporting the hypothesis that OGs mediate at least some responses induced by mechanical damage.

## ARE OGs REGULATORS OF PLANT GROWTH AND DEVELOPMENT?

Dynamic interactions between plant cells depending on the status of pectin in the cell wall are emerging as important regulatory mechanisms of growth and development (Wolf et al., 2012). Because pectin is among the first components that are modified when the wall undergoes physiological remodeling, OGs may be important not only in defense against pathogens, but also under physiological conditions. Over time, OGs have been reported to have effects on plant growth and development. One of the first described effects, i.e., the induction of tomato fruit ripening through the induction of ethylene, was later shown to be mediated by OGs in the size range of DP 4–6 and not 10–15 (Simpson et al., 1998).

Auxins, and in particular indole-3-acetic acid (IAA), are crucial for plant growth and development (Leyser, 2002). Physiological responses to auxins can be antagonized by OGs, as described for the first time by Branca et al. (1988), who showed that auxin-induced elongation in pea stem segments is competitively inhibited by OGs. OGs have been subsequently shown to inhibit auxin-induced root formation in tobacco and *Arabidopsis* leaf explants as well as in thin cell-layer explants (Bellincampi et al., 1993; Savatin et al., 2011) and to induce flower formation in explants that do not normally form organs (Marfà et al., 1991). Moreover, OGs inhibit the stimulation by auxin of the mitotic activity that leads to stomata formation and enhance mean wall thickness of foliar pericycle cells, mainly through cellulose deposition, as well as the number of extra-thick-walled pericycle cells (Altamura et al., 1998). At the molecular level, OGs interfere with the activation of promoters up-regulated by auxin, such as those of the tobacco gene *Nt114* and of the *Agrobacterium rhizogenes rolB* expressed in tobacco (Bellincampi et al., 1996; Mauro et al., 2002). Although OGs do not simply act by inhibiting the action of IAA (Spiro et al., 2002), most of the developmental effects of OGs may be explained with their ability to antagonize auxin responses.

**FIGURE 2:**
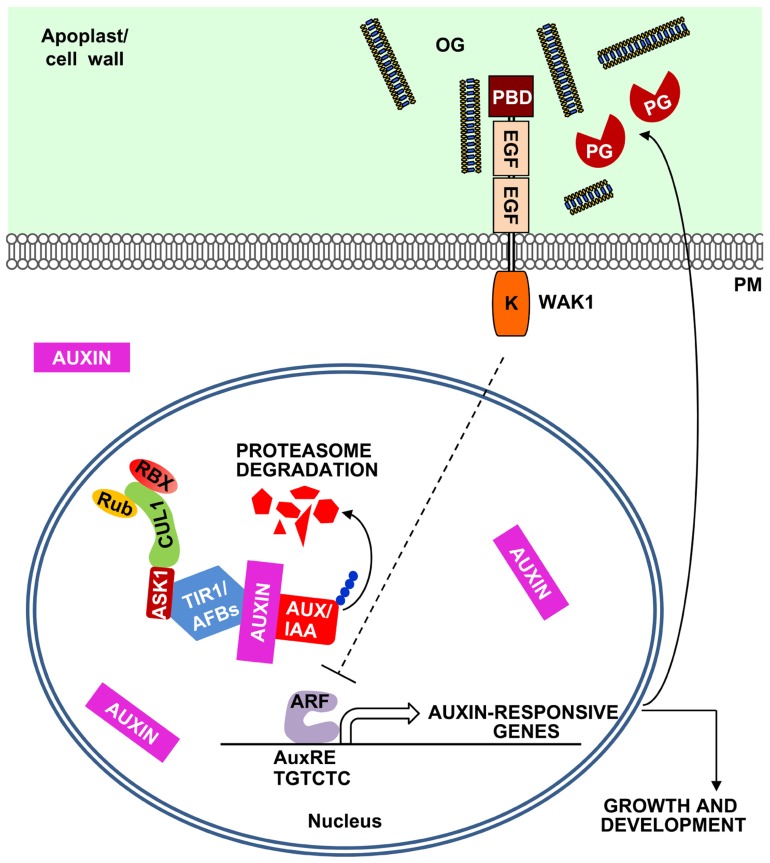
**A model for the OG-mediated negative feedback regulation of the auxin responses.** Plant cells sense auxin through the receptors TIR1/AFBs, F-box proteins that form a SCF E3 ubiquitin ligase complex together with SKP (ASK1) and CULLIN1 (CUL1). This complex is regulated by RUB1 conjugating enzyme (Rub) and RING BOX1 (RBX) proteins and, in the presence of auxin, leads to the ubiquitination of Aux/IAA repressors and their proteasome-mediated degradation. Aux/IAA degradation releases auxin response factors (ARFs) that initiate the transcription of auxin-responsive genes, characterized by the presence of auxin response elements (AuxREs) in their promoters. Auxin also induces the expression of plant PGs and other pectin-degrading enzymes (Laskowski et al., 2006). The action of these enzymes may release in the apoplast OGs that can inhibit auxin-related responses, establishing a negative feedback loop.

In *Arabidopsis*, the transcription of several auxin-induced genes (e.g., *IAA5*, *SAUR16*, and *SAUR-AC*1), as well as the activation of the synthetic auxin-responsive promoter DR5 (Ulmasov et al., 1997) are inhibited by OGs independently of SA, JA, and ethylene and of AtrbohD-mediated H_2_O_2_ accumulation (Savatin et al., 2011). Different elements of the auxin signaling pathway were analyzed as potential targets for OG-mediated inhibition. Auxin acts by binding the F-box protein TRANSPORT INHIBITOR RESPONSE 1 (TIR1) and its homologs AUXIN SIGNALLING F-BOXES (AFBs) and promoting the degradation of the AUXIN/INDOLE-3-ACETIC ACID (Aux/IAA) transcriptional repressors (Gray et al., 2001; Dharmasiri et al., 2005). It was reported that the bacterial elicitor flagellin represses auxin responses in *Arabidopsis* through the induction of a microRNA(miR393) directed against *TIR1/AFB* transcripts (Navarro et al., 2006); this induction, however, occurs only at high doses of the elicitor (Savatin et al., 2011). The antagonism between OGs and auxin does not involve the silencing of *TIR1/AFB* genes, nor requires miR393 activity or post-transcriptional gene silencing (Savatin et al., 2011). Moreover, OG-auxin antagonism also occurs when the auxin-regulated genes are induced by the translation inhibitor cycloheximide, suggesting that OGs may act downstream of Aux/IAA repressors, possibly at the level of the promoter regions of auxin-responsive genes (**Figure 2**).

## CONCLUSION

Oligogalacturonides are very well characterized elicitors of plant defense and are capable of protecting plants against diseases. Their involvement in the local wound response is another interesting feature of OGs. Possibly, these elicitors have a general function of “priming” plant defenses upon cell wall damage that occurs at the early stages of a microbial invasion or during insect attack. OGs may also work as regulators of plant growth and development mainly through their antagonism with auxin. Cell division and elongation are often orchestrated by auxin and require cell wall modifications. It is relevant that auxin induces the expression of PGs and other pectin-degrading enzymes (Laskowski et al., 2006). These enzymes, in turn, may release OGs in the apoplast for a negative feedback regulation of the auxin action. To date, there is no evidence for inhibition of plant PGs by PGIPs; however, the few plant PGs so far characterized have a specific activity much lower than that of the pathogen-secreted enzymes. Thus, the release of OGs during growth and development may not require modulation by PGIPs. The role of OGs as regulators of growth and development is still an interesting speculation, based so far on experiments involving their exogenous applications. Whether OGs accumulate to a significant concentration in intact tissues that are not affected by a microbial infection and whether they act as endogenous regulators of physiological processes is still to be proven.

## Conflict of Interest Statement

The authors declare that the research was conducted in the absence of any commercial or financial relationships that could be construed as a potential conflict of interest.
